# Dr. Mary Walker

**Published:** 1867-08

**Authors:** Roberts Bartholow

**Affiliations:** Late Assistant-Surgeon (Captain) U. S. A.


### ARTICLE IX.

### *Dr. Mary Walker.*

THE notoriety which this mannish female has succeeded in obtaining both during and since the war, has excited a very natural curiosity in reference to her past history. Her true place is so well fixed by a medical officer of the Federal army, that we should injustice to him by an extract. We therefore give his letter entire from the New York Medical Journal for May.

The following communication comes from so respectable a source that we publish it without hesitation :

CINCINNATI, OHIO, January 20th, 1867.

*Editor of* THE NEW YORK MEDICAL JOURNAL.

I observe in the January number of your JOURNAL a page or two devoted to the experiences of Dr. Mary Walker in London. The various newspapers throughout the country have, for the past year or two, contained notices of this person, and a great degree of confusion has arisen as to her real merits, her services in the army, and her position in the medical profession. As I have happened, in the way of official duty, to learn something from this woman herself, I beg to put your readers in posession of my information.

My first observation of Dr. Mary Walker was made at Lincoln General Hospital, Washington, to which she came, in some pretended inspectorial capacity, armed with a pass from Secretary Stanton. The particular function intrusted to Mary Walker seemed to be that of a spy and informer; at all events, she pretended to have power of obtain redress of grievances, and industriously sat about hearing and contriving them. At this period, she was dressed in that hybrid costume which has since become so notorious.

My next encounter with Miss Walker was at Chattanooga. She was sent out by the War Department, through Acting Surgeon-General Barnes, to Assistant Surgeon-General Wood at Louisville. Dr. Wood, it is to be presumed, under instructions from Washington, sent her forward with orders to report to Surgeon Perin, Medical Director of the Army of the Cumberland, she presented herself to Dr. Perin and demanded employment as a Medical Officer. He was not a little astonished at the apparition, and, I may add—I trust without damaging his reputation with the powers that be—indignant that the lives of sick and wounded men should be intrusted to such a medical monstrosity. Before assigning her to duty, which he resolved not to do, he ordered a medical board to examine into her qualifications, as a justification for his decision. I was a member of that Board. *Dr.* Walker presented herself for examination, with a little feminine tremor and confusion, and before settling down to the graver business of the medical examination, tried to propitiate us and secure a favorable report, so that we might take it for granted she possessed the requisite knowledge. She betrayed such utter ignorance of any subject in the whole range of medical science, that we found it a difficult matter to conduct an examination. The Board unanimously reported that she had no more medical knowledge than any ordinary housewife, that she was, of course, entirely unfit for the position of medical officer, and that she might be made useful as a nurse in one of the hospitals.

During the examination, we learned various particulars of her history, which I forbear to mention. She had a diploma, she said—we did not see it—from a "hydropathic institution" at Geneva, N. Y. She had never been, so far as we could learn, within the walls of a medical college or hospital, for the purpose of obtaining a medical education.

The spectacle was both ludicrous and sad. Her pretension, her ignorance, her sex, her unprotected situation, all appealed strongly to our sympathies, and we treated her with the utmost delicacy and consideration. How little ground there was for our feelings of sympathy will appear in the sequel. In a day or two after the examination, she was assigned to a hospital as nurse, but had not entered upon her duties, when an order came from Department Headquarters, sending her to the extreme front! We learned, in a few days, that she was riding about the outposts, and when riding alone, one day, she ventured too far, and was captured and forwarded to Richmond, being treated with considerable rigor, notwithstanding her sex and her claim to the privilege of a medical officer.

It appeared subsequently that this was the design. She was intended as a spy, and went forward to be captured. It was supposed that her sex and *profession* would procure her greater liberties and wider opportunities for observation than were at all possible to other prisoners. The medical staff of the army was made the blind for the execution of this profound piece of strategy by the War Offiice—another instance of the esteem in which medical officers were held by the Hon. Secretary of War.

ROBERTS BARTHOLOW,
Late Assistant-Surgeon (Captain) U. S. A.

